# Supplementary material for: Arterial stiffness and cardiac dysfunction in Hutchinson–Gilford Progeria Syndrome corrected by inhibition of lysyl oxidase
Source: Life Sci Alliance. 2021 Mar 9;4(5):e202000997. doi: 10.26508/lsa.202000997 (PMC8008950; doi:10.26508/lsa.202000997)
Supplement: Supplementary file 1 [file LSA-2020-00997_Supplemental_Data_1.docx]

**Supplemental Methods**

*Carotid artery and aorta immunostaining and histologic analysis.* Isolated arteries were fixed after excision in Prefer (Anatech #414), embedded in paraffin, and 5-µm cross sections were stained. Paraffin-embedded sections (5 µm) of freshly isolated carotid arteries or descending aortas were deparaffinized and hydrated before antigen unmasking (Vector Labs, H3300). Carotid or aortic cross sections were washed in PBS three times before blocking with 2% BSA in PBS for 15 min, incubated overnight at 4^o^C with antibodies directed to p16^INK4A^ (Proteintech, 10883-1-AP; 1:50 dilution), collagen-I (Southern Biotech, 1310-01; 1:400 dilution), collagen-III (Proteintech, 22734-1-AP; 1:400 dilution), collagen-V (Abcam, ab7046; 1:250 dilution), and LOX (Santa-Cruz, sc32409 selective for the LOX isoform; 1:50 dilution). Replicate sections were incubated in parallel with isotype-matched control antibodies. All samples were washed three time with PBS before incubation with a 1:100 dilution of Alexa 594-conjugated isotype-matched secondary antibody (Lifetech donkey anti-goat A11058 or Invitrogen Goat anti-rabbit A11012) for two hours at room temperature. Sections were then washed three times in PBS followed by addition of Dapi (1:1000 dilution in PBS). Slides were briefly washed in PBS and then water before mounting with SlowFade Gold (Thermo, S36936). Results were visualized with a Nikon Eclipse 80i microscope with a QI-Click Qimaging camera. Carotid arteries were imaged at 20x magnification.

Images were quantified using ImageJ. The media of each section was traced using the polygon drawing tool, and its raw integrated intensity was divided by the area of the outlined media to obtain relative fluorescence intensity. The procedure was then repeated for the adventitial layer. Relative fluorescence intensity values were then plotted relative to the mean fluorescence intensity value of the corresponding WT control. Fluorescent intensity values were passed through a Grubbs’ test. Background intensity, as determined from the isotype-matched control antibodies, was negligible (see Fig S5). Results are presented as box plots with Tukey whiskers.

Staining with Hematoxylin (Fisher, SH30-500D) and Eosin (Fisher, SE22-500D) was performed using standard procedures. Apoptosis was determined by immunostaining for cleaved caspase-3 (Cell Signaling Technologies SignalStain Apoptosis IHC Detection kit, #12692S) according to manufactures instructions with tumor xenograft sections as positive controls. To determine calcium deposition, deparaffinized and hydrated sections were incubated with a 5% solution of Alizarin Red S (Sigma, A5533) pH 4.2 for 30 minutes, with calcified bone tissue as positive control. Arterial elastin layers were visualized by autofluorescence using a cyan filter on a Nikon Eclipse 80i Fluorescence microscope.

*Quantification of collagen within elastin folds.* TEM images of vehicle (PBS)-treated or untreated WT and HGPS carotid arteries were taken at 7500 x magnification to visualize the elastin folds (Fig S5; A1). The folds were defined as areas where the elastin invaginates, creating a roughly parabolic shape (Fig. S5; A2). Using the polygon Selection Tool in ImageJ, an area was defined by continuously tracing along the two sides of the parabolic shape; that region was then enclosed by connecting the two apexes with a straight line (Fig. S5; A3). This area was added to the ROI Manager to calculate total area of the elastin fold. To calculate the area within the fold containing collagen, the Paintbrush Tool was used to manually black-out areas containing collagen fibers (Fig. S5; A4). The painted area was isolated with the Threshold Tool (Fig. S5; A5) and added to the ROI manager (Fig. S5; A6). The ratio of collagen/total areas was defined as percent collagen in the elastin fold.

*RT-qPCR of mRNAs and miRs*. Descending aortas from 2-month WT and HGPS mice were isolated and stripped of adventitia. The adventitial and remaining medial/intimal (referred to as medial tissue in the text) tissues were stored separately in RNAlater (Qiagen) at -80^o^C for subsequent analysis. To strip adventitia from the aorta, a cleaned isolated aorta was incubated in 1 mg/ml Type 2 collagenase (Worthington Biochem, LS004174) in Hanks Balanced Salt Solution (Lifetech, 14170-112) for 10 minutes at 37^o^C. The adventitial layer was carefully peeled off before storing the aorta (intimal/medial and adventitial layers separately) in RNAlater. Total RNA was isolated with the RNeasy Fibrous Tissue Mini Kit (Qiagen) according to manufacturer's instructions and using 0.3 ml buffer RLT per aorta. Reverse transcription reactions contained 200-500 ng of total RNA. RNA was extracted from cultures of primary SMCs with TRIzol reagent (Thermofisher 15596026) according to manufacturer’s instruction. microRNA was reverse transcribed from total RNA using the TaqMan microRNA reverse transcription kit (ABI 4366596).

Ten to fifteen percent of the cDNA was subjected to qPCR with the following primer-probe sets from Applied Biosystems: Col1a1 (Mm00801666_g1), Col3a1 (Mm00802300_m1), Col5a1 (Mm00489299_m1); LOX (Mm00495386_m1), LOXL1 (Mm01145738_m1), LOXL2 (Mm00804740), LOXL3 (Mm01184865_m1), LOXL4 (Mm00446385), has-miR-145 (TM: 002278), SnoRNA202 (TM:001232). The primer-probe set for 18S rRNA has been described (16). qPCR samples were analyzed in duplicates. Levels of each tested transcript were normalized to 18S rRNA (for mRNA) or SnoRNA202 (for miRNA), and changes in RNA abundance were calculated using the ddCT method.

*Immunoblotting*. Intact descending aortas (consisting of the adventitia, media and intima) that had been stored in RNAlater (Thermo-Fisher) were chopped into small pieces and sonicated in lysis buffer (50mM Tris-HCl pH8, 250mM NaCl, 2mM EDTA, 1%NP40) containing protease inhibitors (Cell Signaling Technologies 5872S). Lysates were centrifuged for 5 min (4^o^C, 15,000 rpm), and the supernatants were diluted into SDS sample buffer (final concentration of 2% SDS, 10% glycerol, 50mM Tris pH 6.8, 0.01% Bromphenol blue, 1% β-mercaptoethanol) and analyzed by immunoblotting after SDS-PAGE. Approximately 20-30 μg protein was fractionated per sample on an 8% polyacrylamide gel. Lamin A (protein tech 10298), and Focal Adhesion Kinase (BD Transduction Labs 610088), and Lysyl Oxidase (abcam ab31238) antibodies were used at a 1:300 dilution.

*Lysyl Oxidase Activity Assay.* Intact descending aortas were isolated, flash frozen in liquid nitrogen and stored at -80^o^C prior to the analysis of LOX activity. Thawed aortas were chopped into small pieces and sonicated in 0.2ml PBS three times for 15-20 seconds each and stored immediately on ice. Lysates were centrifuged at 14,000 x *g* for 5 min, and 50 μl of each supernatant was analyzed using the Abcam Lysyl oxidase activity assay kit (ab112139) according to manufacturer’s instructions. Samples were run in technical duplicates. Relative fluorescent intensity values were normalized to the total protein concentration of each lysate as determined using BioRad protein concentration reagent (Biorad 5000006).

*Myograph data analysis.* Measurements of intraluminal pressure, force, and outer diameter were converted into stress-stretch curves using equations 1–4 where *l* and *L*=loaded and unloaded vessel lengths, respectively (µm), *a_i_* and *A_i_*=loaded and unloaded inner radii, respectively (µm), *a_o_* and *A_o_*=loaded and unloaded outer radii, respectively (µm), *h* and *H* = loaded and unloaded vessel wall thickness, respectively (µm), *P*= intraluminal pressure (mm Hg), and *f_T_*=axial force (nN). Vessel wall thickness was calculated in the post-test analysis as described (15) with the standard assumption that the sample was incompressible.

Equation 1: Axial stretch $(\text{λ}_{\text{z}}\text{)=}\frac{\text{l}}{\text{L}}$

Equation 2: Axial stress $(\text{σ}_{\text{z}}\text{)=}\text{ }\frac{\text{P}\text{a}^{\text{2}}\text{π + }\text{f}_{\text{T}}}{\text{πh(2a + h)}}$

Equation 3: Circumferential stretch $(\text{λ}_{\text{θ}}\text{)=}\frac{\text{a + h/2}}{\text{A + H/2}}$

Equation 4: Circumferential stress $(\text{σ}_{\text{θ}}\text{)=}\frac{\text{Pa}\text{i}}{\text{h}}$

Axial stress-stretch curves show means ± SD. Circumferential stress-stretch curves as well as inner radius and wall thickness results were determined from triplicate determinations per sample and are therefore presented as means ± SE.

To estimate tissue stiffness, axial and unloaded circumferential stresses and stretches for each tested vessel were fit to an exponential function (y=a^bx^) using Matlab (R2020a, Curve Fitting Tool). The derivative of each vessels exponential fit was then used to plot the tangent modulus as a function of stretch for each individual vessel. Tangent modulus values were passed through the Grubbs’ test and one mouse was eliminated as an outlier. These results were then averaged and plotted as the tangent modulus graphs. The adjusted R^2^ values identified in MatLab for each fit were assessed to be greater than 0.95 in order to be included in the mean tangent modulus curves.

*Echocardiography*. Ultrasound examination of the left ventricle was performed using a Fujifilm VisualSonics Ultrasound System (VisualSonics Inc, Toronto, ON, Canada) and using MS400 (18-38 MHZ) transducer. Mice were lightly anesthetized with an i.p. injection of 0.005 ml/g of 2% Avertin (2,2,2-Tribromoethanol, Sigma-Aldrich, St. Louis, MO). Hair was removed from the anterior chest using chemical hair remover (Nair), and the animals were placed on a warming pad in a left lateral decubitus position to maintain normothermia (37°C), monitored by a rectal thermometer. Ultrasound gel was applied to the chest. Care was taken to maintain adequate contact while avoiding excessive pressure on the chest. Left ventricular (LV) systolic function: Two-dimensional long-axis, short-axis M-Mode images were obtained. LV diastolic function: Transmitral inflow pattern and tissue Doppler were obtained in modified 4 chamber apical view. After completion of the imaging studies, mice were allowed to recover from anesthesia and returned to their cages. M Mode Images were analyzed for LV structure and function related parameters and Pulse wave and tissue Doppler images were analyzed for diastolic function related parameters using Vevo Lab software (Visual Sonics Inc, Toronto, ON, Canada).

*Aortic Catheterization*. Closed-chest cardiac catheterization was performed under isoflurane (1.5-4%) by ligating the right carotid artery and advancing a 1F Millar catheter (SPR-1000) into the ascending aorta, where it was secured. Measurements were taken using Millar MPVS Ultra and ADInstruments LabChart for approximately 5 minutes after catheter insertion. The temperature of the animal was maintained between 36.5-37.5 degrees Celsius.

*Cell culture, viral infection and RNAi*. Primary mouse SMCs were isolated from the descending aortas of 2-month old WT and HGPS male mice and prepared by explant culture as described (16). SMCs were cultured in growth medium [1:1 Dulbecco’s modified Eagle’s medium (DMEM)/Ham’s F-12 supplemented with 2mM L-glutamine and 20mM HEPES, pH7.4] with 20% FBS. Cells were passaged at near confluence with trypsin/EDTA and used between passages 2-4. Near confluent, asynchronous SMCs were infected with adenoviruses encoding LacZ (control) or miR-145 (AdmiRa-has-miR-145-5p; ABM #mh0185) at a MOI of 600 in growth medium and incubated overnight. The cells were incubated for 72 hours in fresh growth medium before sample collection. Infection efficiency of the miR-145 adenovirus was ~70% as determined by expression of co-transcribed GFP. siRNA-mediated knock-down of Lamin A in near-confluent WT SMCs was performed using Lipofectamine RNAiMAX Transfection Reagent (Thermofisher #13778100) in OPTI-MEM with final siRNA concentrations of 150 nM (Thermofisher siRNA ID #s69252, #s69253, #s69254). A non-specific siRNA (Ambion 4390843) was used as control. After 4 hours of siRNA transfection, cells were switched to fresh growth medium. Total RNA or protein was collected 72 hours after transfection using TRIzol reagent (for RNA analysis) or immunoblotting lysis buffer (see above), respectively.

**Supplemental References:**

1. Engström K, Willén H, Kåbjön-Gustafsson C, Andersson C, Olsson M, Göransson M, Järnum S, Olofsson A, Warnhammar E, aman P. The myxoid/round cell liposarcoma fusion oncogene FUS-DDIT3 and the normal DDIT3 induce a liposarcoma phenotype in transfected human fibrosarcoma cells. Am J Pathol. 2006. 168:1642-53.

2. Johnston IM, Spence HJ, Winnie JN, McGarry L, Vass JK, Meagher L, Stapleton G, Ozanne BW. Regulation of a multigenic invasion programme by the transcription factor, AP-1: re-expression of a down-regulated gene, TSC-36, inhibits invasion. Oncogene. 2000. 19:5348-58

3. Adamopoulos, C., Piperi, C., Gargalionis, A. N., Dalagiorgou, G., Spilioti, E., Korkolopoulou, P., … Papavassiliou, A. G. (2016). Advanced glycation end products upregulate lysyl oxidase and endothelin-1 in human aortic endothelial cells via parallel activation of ERK1/2-NF-κB and JNK-AP-1 signaling pathways. *Cellular and Molecular Life Sciences*, *73*(8), 1685–1698.

4. Tenti, P., & Vannucci, L. (2020, February 1). Lysyl oxidases: linking structures and immunity in the tumor microenvironment. *Cancer Immunology, Immunotherapy*. Springer.

5. O'Connell BC, Cheung AF, Simkevich CP, Tam W, Ren X, Mateyak MK, Sedivy JM. A large scale genetic analysis of c-Myc-regulated gene expression patterns. J Biol Chem. 2003. 278:12563-73.

6. Bogachek MV, Chen Y, Kulak MV, Woodfield GW, Cyr AR, Park JM, Spanheimer PM, Li Y, Li T, Weigel RJ. Sumoylation pathway is required to maintain the basal breast cancer subtype. Cancer Cell. 2014. 25:748-61.

7. Cai Y, Balli D, Ustiyan V, Fulford L, Hiller A, Misetic V, Zhang Y, Paluch AM, Waltz SE, Kasper S, Kalin TV. Foxm1 expression in prostate epithelial cells is essential for prostate carcinogenesis. J Biol Chem. 2013. 288:22527-41.

8. Park HJ, Gusarova G, Wang Z, Carr JR, Li J, Kim KH, Qiu J, Park YD, Williamson PR, Hay N, Tyner AL, Lau LF, Costa RH, Raychaudhuri P. Deregulation of FoxM1b leads to tumour metastasis. EMBO Mol Med. 2011. 3:21-34.

9. Wang V, Davis DA, Haque M, Huang LE, Yarchoan R. Differential gene up-regulation by hypoxia-inducible factor-1alpha and hypoxia-inducible factor-2alpha in HEK293T cells. Cancer Res. 2005. 65:3299-306.

10. Elvidge GP, Glenny L, Appelhoff RJ, Ratcliffe PJ, Ragoussis J, Gleadle JM. Concordant regulation of gene expression by hypoxia and 2-oxoglutarate-dependent dioxygenase inhibition: the role of HIF-1alpha, HIF-2alpha, and other pathways. J Biol Chem. 2006. 281:15215-26.

11. Schietke R, Warnecke C, Wacker I, Schödel J, Mole DR, Campean V, Amann K, Goppelt-Struebe M, Behrens J, Eckardt KU, Wiesener MS. The lysyl oxidases LOX and LOXL2 are necessary and sufficient to repress E-cadherin in hypoxia: insights into cellular transformation processes mediated by HIF-1. J Biol Chem. 2010. 285:6658-69.

12. Pawlus MR, Wang L, Hu CJ. STAT3 and HIF1α cooperatively activate HIF1 target genes in MDA-MB-231 and RCC4 cells. Oncogene. 2014. 33:1670-9.

13. Mathison A, Grzenda A, Lomberk G, Velez G, Buttar N, Tietz P, Hendrickson H, Liebl A, Xiong YY, Gores G, Fernandez-Zapico M, Larusso NF, Faubion W, Shah VH, Urrutia R. Role for Krüppel-like transcription factor 11 in mesenchymal cell function and fibrosis. PLoS One. 2013. 8:e75311.

14. Klein EA, Yung Y, Castagnino P, Kothapalli D & Assoian RK. Cell Adhesion, Cellular Tension, and Cell Cycle Control. In *Methods in enzymology*. 2007. pp.155–175.

15. Brankovic S, Hawthorne EA, Yu X, Zhang Y & Assoian RK. MMP12 preferentially attenuates axial stiffening of aging arteries. *J. Biomech. Eng.* 2019.

16. Cuff CA, Kothapalli D, Azonobi I, Chun S, Zhang Y, Belkin R, Yeh C, Secreto A, Assoian RK, Rader DJ & Puré E. The adhesion receptor CD44 promotes atherosclerosis by mediating inflammatory cell recruitment and vascular cell activation. *J. Clin. Invest.* 2001. 108, 1031–1040.
